# Brown Bread

**Published:** 1887-10

**Authors:** 


### Brown Bread.

Dr. Geo. D. Hays, of the New York Post Graduate School, writing in the *Quarterly Bulletin*, says: "We have long been accustomed to hear that many of the evils of modern life owe their origin to our choice of *white* flour. That this is not so an examination of the wheat-berry will show. This has five coats —an epi-, messo-, and endocarp, an episperm and a tegmen. The three outer ones have no value whatever as nutriment. Within the episperm is a layer of gluten-cells, chiefly albuminoids, and finally in the endosperm, which constitutes the bulk of the grain, we find starch mixed with albuminoid cells. In the old process of milling the perisperm (the part within the episperm) was, on account of its close attachment to the inner husk, largely carried away, leaving the bolted flour the poorer for its loss. Hence the vegetarian, Sylvester Graham, whose name is applied to bread made from unbolted flour, was correct in his time in saying such bread contained the most nutriment. The present gradual reduction process saves this portion of the wheat. The bran itself is composed of woody fibre, and contains absolutely no nutriment. It may have a mechanical value in those of a constipated tendency, but this is all. The wheat loaf and the white flour contain a much larger percentage of phosphates and gluten than the Graham loaf or unbolted flour."

